# SIRT3 in Cardiac Physiology and Disease

**DOI:** 10.3389/fcvm.2016.00038

**Published:** 2016-10-13

**Authors:** Christoph Koentges, Christoph Bode, Heiko Bugger

**Affiliations:** ^1^Division of Cardiology and Angiology I, Heart Center Freiburg University, Freiburg, Germany

**Keywords:** sirtuin 3, mitochondria, heart failure, ischemia–reperfusion injury

## Abstract

Functional defects in mitochondrial biology causally contribute to various human diseases, including cardiovascular disease. Impairment in oxidative phosphorylation, mitochondrial oxidative stress, and increased opening of the mitochondrial permeability transition pore add to the underlying mechanisms of heart failure or myocardial ischemia–reperfusion (IR) injury. Recent evidence demonstrated that the mitochondrial NAD^+^-dependent deacetylase sirtuin 3 (SIRT3) may regulate these mitochondrial functions by reversible protein lysine deacetylation. Loss of function studies demonstrated a role of impaired SIRT3 activity in the pathogenesis of myocardial IR injury as well as in the development of cardiac hypertrophy and the transition into heart failure. Gain of function studies and treatment approaches increasing mitochondrial NAD^+^ availability that ameliorate these cardiac pathologies have led to the proposal that activation of SIRT3 may represent a promising therapeutic strategy to improve mitochondrial derangements in various cardiac pathologies. In the current review, we will present and discuss the available literature on the role of SIRT3 in cardiac physiology and disease.

## Introduction

The sirtuin family of protein deacetylases consists of seven mammalian members (SIRT1-7), which are orthologs of the silent information regulator two (Sir2) that was first identified in yeast almost two decades ago (1, 2). Sirtuins are classified as class III histone deacetylases, requiring NAD^+^ as a cosubstrate, which acts as an acceptor molecule for the acetyl group removed from the target protein (3). The need of NAD^+^ as a cosubstrate links activity of sirtuins tightly to the cellular energy status making them potential redox sensors (4–6). SIRT3, SIRT4, and SIRT5 are primarily localized inside the mitochondria, and SIRT3 possesses the most robust deacetylase activity of all mitochondrial sirtuins (7, 8). Although named histone deacetylases, numerous non-histone protein targets have been identified using large-scale proteomic analyses, also including proteins localized outside of mitochondria (9, 10). Deacetylation targets of SIRT3 include metabolic enzymes involved in fatty acid oxidation (FAO) and glucose oxidation (GLOX), protein subunits of the electron transport chain (ETC), as well as enzymes involved in oxidative stress defense and mitochondrial integrity (7, 10–21).

SIRT3 is highly expressed in tissues with high metabolic turnover and mitochondrial content, including the heart and its role in cardiac physiology and pathology have been increasingly investigated in recent years (8, 17, 22–29). Under normal physiologic conditions, about 95% of ATP used to maintain cardiac pump function is regenerated within mitochondria by the oxidation of fatty acids and glucose, with some contribution from other substrates as well (30). Mitochondrial derangements such as defects in substrate oxidation and oxidative phosphorylation, mitochondrial oxidative stress, and increased sensitivity for opening of the mitochondrial permeability transition pore (mPTP) are major mechanisms underlying clinically important cardiac pathologies, such as heart failure and ischemia–reperfusion (IR) injury (31–36). A number of studies provided evidence that SIRT3 regulates these mechanisms in the heart. In this review, we will present and discuss the current knowledge on the role of SIRT3 in cardiac physiology and disease and potential therapeutic strategies aiming at modulating SIRT3 activity.

## SIRT3 in Cardiac Physiology

### SIRT3 and Cardiac Function

The impact of SIRT3 in the regulation of cardiac function has been evaluated in a number of studies using mice with global deletion of SIRT3 (SIRT3^−/−^) (13, 17, 22–26, 29). Using echocardiography, fractional shortening (FS) and ejection fraction (EF) were not different between SIRT3^−/−^ and wild-type (WT) mice at 8 weeks of age (17, 23, 25, 28, 29, 37). However, in older animals (24 weeks of age), SIRT3^−/−^ mice displayed a decrease in EF and FS, accompanied by increased systolic and diastolic diameters and volumes of the left ventricle (LV) indicative of LV dilation (23, 28). Thus, SIRT3^−/−^ mice seem to develop an accelerated age-related decline in cardiac contractile function (23, 28). Interestingly, when perfused in the isolated working heart model, even 8-week-old SIRT3^−/−^ mice display contractile defects, such as a decrease in cardiac power, cardiac output, and developed pressure (22, 23). Possibly, contractile dysfunction could not be observed in young mice *in vivo* but only *ex vivo* since neurohormonal compensatory mechanisms may only be present in the live animal (e.g., renin–angiotensin–aldosterone–system, autonomic nervous system, changes in vasomotor and/or heart rate), but absent in isolated perfused hearts. With increasing age, the progress in cardiac remodeling due to SIRT3 deficiency (e.g., increased fibrosis, LV dilation) may dominate these compensatory mechanisms leading to obvious cardiac dysfunction detectable by echocardiography. Furthermore, aging-associated abnormalities in mitochondrial function, such as increased oxidative stress, mutations of mitochondrial DNA, and decreased mitochondrial respiration may predispose for a stronger impact of SIRT3 deficiency on overall mitochondrial function and integrity in older versus younger mice (38). Interestingly, one study did not observe impairment in contractile function in 24- to 26-week-old mice in isolated working hearts (24). In this study though, hearts were perfused in the presence of insulin and higher concentrations of fatty acids compared to Koentges and colleagues (23). The impact of insulin and substrate availability on cardiac function and energetics in SIRT3^−/−^ mice remains to be determined in more detail.

Further evidence supporting the proposal of SIRT3 being required to maintain cardiac function comes from studies that increased cardiac energy demand by increasing cardiac workload. Following transverse aortic constriction (TAC), SIRT3^−/−^ mice display an accelerated development of contractile dysfunction with a more pronounced decrease in EF and a more pronounced increase in endsystolic volume (23). Under these conditions, SIRT3^−/−^ mice develop a greater degree of cardiac fibrosis and a more pronounced increase in cardiomyocyte hypertrophy (13, 17, 23, 25). Vice versa, Sundaresan et al. could prevent cardiac contractile dysfunction and attenuate the extent of fibrosis after angiotensin II (AngII) infusion by cardiomyocyte-specific overexpression of SIRT3 (17). Interestingly, TAC-induced contractile dysfunction with concomitant reduction of SIRT3 expression in WT mice was rescued by reinduction of SIRT3 expression by administration of honokiol, a compound derived from magnolian trees (39). Taken together, these studies suggest that SIRT3 seems to be required to maintain cardiac contractile function.

### Targets and Pathways Regulated by SIRT3

Cardiac function is tightly linked to the continuous delivery of energy-rich phosphates, 95% of which are generated by mitochondrial oxidative phosphorylation. Quite a number of studies reported that SIRT3 regulates energy metabolism in various tissues, including the heart. Hirschey et al. reported decreased rates of palmitate oxidation in cardiac tissue extracts from SIRT3^−/−^ mice, and Ahn et al. reported lower cardiac ATP levels in these mice (16, 40). These findings were confirmed in isolated working heart experiments showing decreased rates of palmitate and GLOX and lower rates of myocardial O_2_ consumption in SIRT3^−/−^ mice (23). In addition, isolated cardiac mitochondria showed lower rates of ATP production, resulting in lower ATP/AMP ratios, and thus decreased cellular energy charge (23). Impairment in mitochondrial function may be the consequence of hyperacetylation of several SIRT3 target enzymes in the absence of SIRT3. SIRT3 target enzymes range through almost all substrate oxidation and ATP-generating processes from pyruvate import (mitochondrial pyruvate carrier), TCA cycle activity and its anaplerosis (acetyl-CoA synthetase, aconitase, glutamate dehydrogenase, isocitrate dehydrogenase), β-oxidation of fatty acids (long-chain acyl-CoA dehydrogenase), to oxidative phosphorylation (Ndufa9, succinate dehydrogenase and ATP synthetase subunit a) and ATP translocation (adenine nucleotide translocase) (7, 11, 12, 14, 16, 19, 20, 23, 40, 41). Most of the described targets of SIRT3 are thought to be activated by SIRT3-mediated protein deacetylation.

Since SIRT3 activity is directly linked to the NAD^+^/NADH ratio, SIRT3 may serve as a metabolic sensor. In situations of high energy demand, the NAD^+^/NADH ratio rises and subsequently increases SIRT3 activity, which may translate into deacetylation of metabolic enzymes and thus acceleration of metabolic pathway flux and energy production. A dysbalance between energy demand and supply may occur during physical exercise or changes in blood pressure or during the 24-h fasting and feeding cycle, when NAD^+^ levels oscillate in a circadian fashion (42). Given the fact that SIRT3 drives mitochondrial substrate oxidation and thus ETC flux, it is not surprising that SIRT3 participates in the detoxification of reactive oxygen species (ROS). Manganese superoxide dismutase (MnSOD), a major intramitochondrial antioxidant enzyme, has been reported to be a deacetylation target of SIRT3, and MnSOD expression is also controlled by SIRT3-mediated nuclear translocation of the transcription factor forkhead-box-protein 3a (Foxo3a) (17, 43, 44). SIRT3 may, thereby, protect the heart from oxidative damage induced by increased production of ROS, which may result as byproducts of accelerated ETC flux.

Of note, the assumption that a lower degree of lysine acetylation is connected to a higher activity of the affected enzyme should be drawn carefully. Although most of the described deacetylation targets of SIRT3 are activated by deacetylation, Fernandes et al. recently reported that increased acetylation of aconitase 2 (ACO2) increases ACO2 enzymatic activity (12). The authors argue that lower expression of SIRT3 induced by high-fat diet feeding may increase ACO2 activity, which may increase cytosolic citrate levels and subsequently inhibit acetyl-CoA-carboxylase, resulting in lower malonyl-CoA levels and subsequent derepression of carnitine-palmitoyl transferase (12). Lower SIRT3 levels would, thereby, adapt mitochondrial β-oxidation to higher levels of circulating fatty acids. In another study, Alrob et al. reported decreased SIRT3 protein levels after feeding a high-fat diet, which was associated with higher acetylation levels of long-chain acyl-CoA dehydrogenase (LCAD) and 3-hydroxyacyl-CoA dehydrogenase leading to higher enzyme activity and FAO rates (24). Of interest, increased acetylation of LCAD has been reported to both increase and decrease its activity. One possible explanation for these conflicting results may be differences in the acetylation pattern of LCAD, which may differentially affect enzymatic activity. For example, p53 possesses 13 potential acetylation sites in different domains (45). Mathematically, 6,227,020,800 different combinations of lysine acetylations would be possible, which may change p53 protein function. To date, only few data are available on specific acetylation patterns induced by SIRT3 or SIRT3 deficiency and the effect on target enzyme activity.

### SIRT3 Expression in the Heart

SIRT3 is highly expressed in organs with high metabolic turnover, e.g., heart, liver, brain, and kidney (40). In the heart, SIRT3 expression responds to changes in energy demand (16, 23, 25, 27, 29, 39, 46). In mice, myocardial SIRT3 expression was increased following TAC or infusion of isoproterenol or AngII, probably to match oxidative capacity to increased demand for ATP regeneration in hypertrophied hearts (17, 23). In contrast though, when hypertrophy progresses to heart failure, or in doxorubicin-induced heart failure, expression of SIRT3 was decreased (25, 27, 47). Similarly, SIRT3 expression was decreased in human failing hearts (48, 49). Thus, the downregulation of SIRT3 may rather be maladaptive in the failing heart and may exaggerate mitochondrial dysfunction and oxidative stress.

A downregulation of SIRT3 was also observed in hearts of mice fed a high-fat diet, in obese, and type 2 diabetic *db/db* mice, and in rodents with streptozotocin-induced type 1 diabetes, suggesting that downregulation of SIRT3 in the heart may also be a hallmark of the metabolic syndrome and diabetes mellitus, although a downregulation of SIRT3 was not observed in every single study (24, 28, 50–52). Similar to failing hearts, SIRT3 downregulation may also contribute to myocardial mitochondrial dysfunction and oxidative stress in diabetes, but may also contribute to reduced capillary density in diabetic hearts (28, 50, 53).

Upstream signaling that regulates SIRT3 expression is poorly understood, but may involve the master regulator of mitochondrial biogenesis and gene expression, peroxisome proliferator-activated receptor gamma coactivator 1 α (PGC-1α). Kong et al. demonstrated that PGC-1α regulates SIRT3 expression *via* binding of ERRα to an ERR response element on the SIRT3 promoter in C2C12 myotubes (54). In skeletal muscle, dihydromyricetin improved insulin sensitivity by induction of autophagy *via* activation of the AMPK-PGC-1α-Sirt3 signaling pathway (55). Thus, SIRT3 expression may be regulated by PGC-1α, and reduced expression of SIRT3 in heart failure may thus be related to downregulation of PGC-1α in failing hearts (48, 49, 56). This hypothesis, however, remains to be tested experimentally.

## SIRT3 in Cardiac Disease

### SIRT3 in Hypertrophy and Heart Failure

Increasing evidence suggests a causative role of impaired SIRT3 activity in a number of cardiac pathologies, including cardiac hypertrophy and heart failure. SIRT3^−/−^ mice spontaneously develop cardiac hypertrophy with increasing age (13, 17, 23, 29). The hypertrophic response to pressure overload (induced by TAC), treatment with AngII, or infusion of isoproterenol is more pronounced in SIRT3^−/−^ mice compared with non-transgenic controls (13, 17, 23, 29). The exacerbated hypertrophy is accompanied by a more pronounced increase in interstitial fibrosis as well. Conversely, overexpression or pharmacological activation of SIRT3 was able to ameliorate or even block cardiac hypertrophy and interstitial fibrosis in response to pressure overload or hypertrophy agonist treatment (17, 29, 47).

Several mechanisms may contribute to the beneficial and deleterious effects of SIRT3 activation and deletion, respectively. SIRT3 has numerous targets in mitochondrial energy metabolic pathways, which are hyperacetylated in mice lacking SIRT3. A coordinated slowing of FAO, GLOX, the TCA cycle, and oxidative phosphorylation with subsequent myocardial energy depletion due to decreased SIRT3 activity may, thus, be a plausible mechanism contributing to contractile dysfunction and impaired adaptation to increased energy demands (23, 40). Another important mechanism may be an imbalance between ROS production and expression and activity of antioxidative enzymes, resulting in oxidative stress. SIRT3^−/−^ mice show signs of increased oxidative stress, e.g., increased levels of 4-HNE and TBARS (15, 23). Vice versa, overexpression of SIRT3 prevents ROS accumulation during hypertrophy, probably by preventing a drop in catalase and MnSOD expression after AngII infusion, likely due to an increase in FOXO3a signaling (17). In addition, SIRT3 is capable of deacetylating MnSOD, thereby increasing its activity and attenuating oxidative stress (17). Protection from oxidative stress may also attenuate activation of the ROS-sensitive MAPK/ERK and PI3K/AKT signaling pathways, which are known to play a major role in the development of cardiac hypertrophy (57). Cytosolic ROS may be of minor importance though, since ROS scavenging by treatment with the antioxidant 4-hydroxy-2,2,6,6-tetramethylpiperidin-1-oxyl (TEMPOL) did not improve contractile deficits in hearts of SIRT3^−/−^ mice (23). Increased fibrosis in aged SIRT3^−/−^ mice and following TAC may be related to a disinhibition of transforming growth factor β1 (TGF-β1) signaling and hyperacetylation of glycogen synthase kinase 3β (GSK3β) by SIRT3 deficiency, resulting in increased expression of profibrotic genes (58).

The above mentioned mechanisms may well contribute to mitochondrial dysfunction typically observed in failing hearts. Indeed, SIRT3 expression is reduced and global mitochondrial protein lysine acetylation is increased in rodent models of heart failure, suggestive of impaired SIRT3 activity (48). Reduced SIRT3 expression may be the consequence of PGC-1α downregulation, suggesting that impaired PGC-1α-SIRT3 signaling may impair mitochondrial function by several mechanisms, including inhibition of substrate oxidation due to enzyme hyperacetylation, increased oxidative stress, and impaired OXPHOS expression due to impaired PGC-1α signaling *per se*. Another mechanism which may impair SIRT3 activity in failing hearts may be NAD^+^ depletion or a decrease in the NAD^+^/NADH ratio. Poly(ADP-ribose)-polymerase 1 (PARP-1) is a DNA repair enzyme which is overactivated in failing hearts, and PARP-1 also uses NAD^+^ as a cosubstrate (59). Overactivation of PARP-1 leads to cellular NAD^+^ depletion, potentially resulting in impaired SIRT3 activity (59, 60). A decrease in the NAD^+^/NADH ratio may also be caused by preexisting mitochondrial dysfunction *per se*. Karamanlidis et al. demonstrated a decrease in the NAD^+^/NADH ratio in a mouse model with complex I (Ndufs4 subunit) deficiency, resulting in heart failure (5). Since protein levels of SIRT3/4/5 were unchanged, they proposed that inhibition of SIRT3 activity by a low NAD^+^/NADH ratio may have caused hyperacetylation of mitochondrial proteins, which would further impair the preexisting mitochondrial dysfunction due to complex I deficiency. Indeed, restoration of the NAD^+^/NADH ratio by treatment with β-nicotinamide mononucleotide (NMN) decreased mitochondrial protein acetylation in mice with complex I deficiency (5). Thus, both, reduced expression of SIRT3 and a decrease in NAD^+^ availability may contribute to impaired SIRT3 activity in heart failure.

### SIRT3 in IR Injury

Mitochondria play a pivotal role in the pathogenesis of myocardial IR injury. Impaired mitochondrial respiration, mitochondrial oxidative stress, and increased opening of the mPTP determine myocardial infarct size and recovery of contractile function following an ischemic episode (61, 62). Porter et al. reported pronounced impairment in recovery of contractile function and an increase in myocardial infarct size following ischemia in 28-week-old SIRT3^+/−^ mice compared with WT controls (26). Regarding the mechanisms, SIRT3^+/−^ mice showed a decrease in complex I activity, suggesting impairment in mitochondrial function, and a decrease in MnSOD activity, suggesting impaired oxidative stress defense (26). In addition, Hafner et al. reported increased sensitivity for mPTP opening in SIRT3^−/−^ mice, likely due to deacetylation of lysine 166 of cyclophilin D, a critical component of the mPTP (13). Since mitochondrial NAD^+^ levels drop during myocardial IR in WT mice, SIRT3 activity may be impaired during IR and, thus, contribute to IR injury in the heart (63).

Of interest and in contrast to SIRT3 heterozygous mice, SIRT3^−/−^ mice showed no additional impairment in recovery of contractile function following global no-flow ischemia in the isolated working heart model (22). In addition, rates of mitochondrial O_2_ consumption, 4-HNE levels, and Ca^2+^-induced mitochondrial swelling were not different to WT controls undergoing IR (22). However, SIRT3^−/−^ mice were younger (8 weeks of age) compared with 6-month-old mice in the study by Porter and colleagues (26). Thus, these results suggest an age-dependent increase in susceptibility for IR injury in SIRT3^−/−^ mice. In fact, the extent of IR injury generally increases with age, accompanied by abnormalities of mitochondrial biology, such as increased ROS production, decreased mitochondrial cristae density, and impairment in oxidative phosphorylation and ETC complex activities (64–71). Thus, it is tempting to speculate that lack of SIRT3 *per se* may not necessarily aggravate IR injury, but with increasing age, the presence of additional defects in mitochondrial function may ultimately increase the overall vulnerability of SIRT3^−/−^ mice for cardiac damage following myocardial IR. Of note, it has been reported that the expression of SIRT3 decreases with increasing age in various organs in mice (kidney, brain, aortic valve tissue), and that NAD^+^ levels also drop with increasing age in *C. elegans* and liver and skeletal muscle tissue in mice (37, 72–76). Thus, a combination of decreased SIRT3 expression and activity may contribute to a greater extent of IR injury with increasing age.

## SIRT3 as a Potential Therapeutic Target

**Figure 1:**
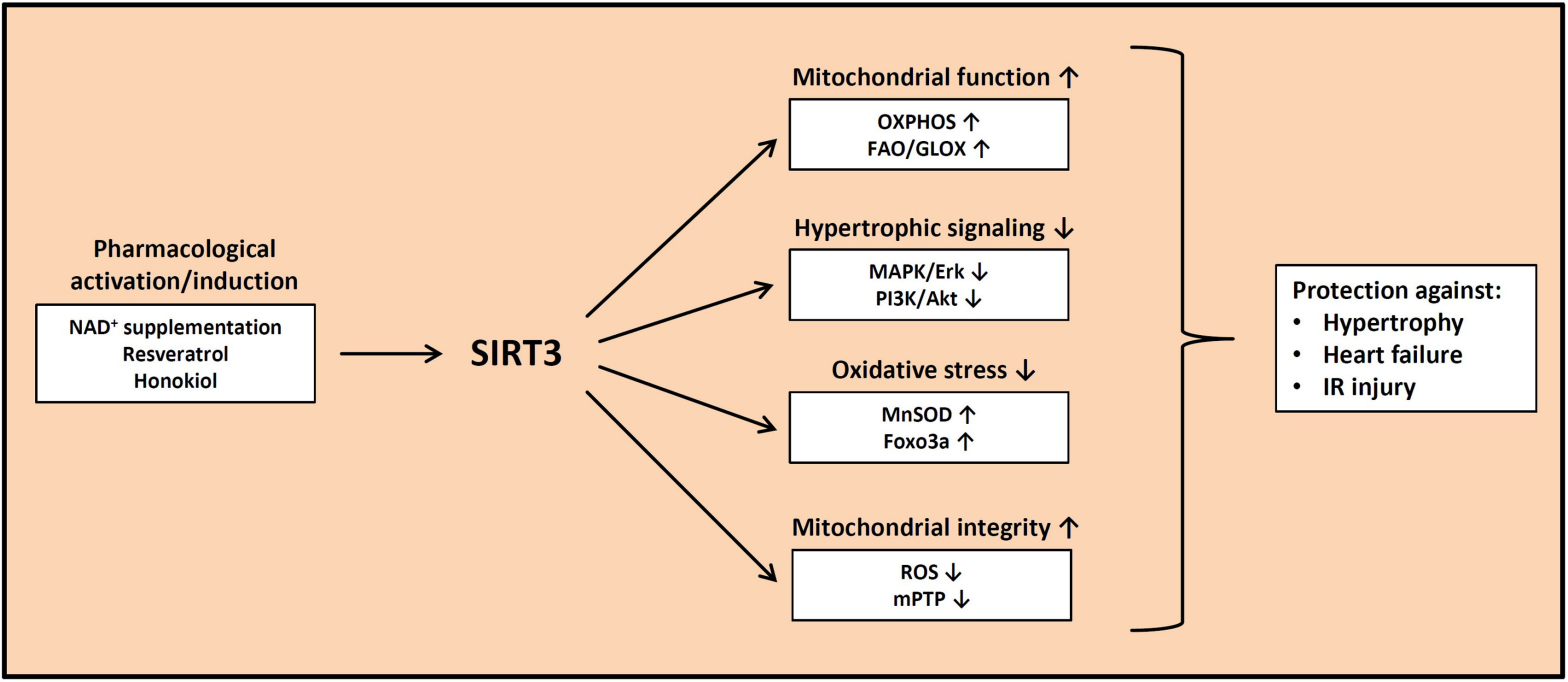
**Beneficial effects of increased SIRT3 activity**. Pharmacological activation and/or induction of SIRT3 expression may protect against cardiac hypertrophy, heart failure, and ischemia–reperfusion injury by improving mitochondrial substrate oxidation, decreasing prohypertrophic signaling, attenuating oxidative stress, and maintaining mitochondrial integrity. OXPHOS, oxidative phosphorylation; FAO, fatty acid oxidation; GLOX, glucose oxidation; MnSOD, manganese superoxide dismutase; ROS, reactive oxygen species; mPTP, mitochondrial permeability transition pore.

Based on the evidence presented in the previous paragraphs, impaired SIRT3 activity may play an important role in the pathogenesis of cardiac hypertrophy, heart failure, and IR injury. Thus, different pharmacological approaches have been pursued to either increase SIRT3 expression or to increase SIRT3 activity by raising NAD^+^ levels (Figure 1). Honokiol is a polyphenol that belongs to a class of neolignan biphenols and that has a high bioavailability. It is derived from bark of magnolian trees and has been widely used in traditional medicine in China, Korea, and Japan. Honokiol may exert a large number of effects, including anti-inflammatory effects, anti-apoptotic effects, antioxidant effects, anti-tumorigenic effects, may activate PPARγ signaling, and interferes with MAPK and PI3K/Akt signaling, among several other effects (77, 78). Pillai et al. recently showed that the administration of honokiol is capable of reversing cardiac hypertrophy and of rescuing contractile dysfunction following TAC in mice (39). The authors showed that honokiol was present within mitochondria and enhanced SIRT3 expression nearly two-fold, accompanied by reduced deacetylation of mitochondrial SIRT3 target proteins (39). Another natural biphenol that is contained in the skin of grapes, blueberries, and raspberries and that may modulate SIRT3 activity, is resveratrol (RSV). RSV exerts multiple effects on the cardiomyocyte, including AMPK activation, induction of autophagy, inhibition of ROS production, vasorelaxation, inhibition of atherosclerosis, anti-inflammatory effects, and boosting mitochondrial biogenesis, using many different signaling pathways outlined in more detail elsewhere (79, 80). Regarding sirtuins, RSV was originally reported to activate SIRT1 and is thought to decrease the risk of heart disease (79). Chen et al. recently reported though that RSV prevented cardiac hypertrophy in response to hypertrophic stimuli in WT mice, and this protective effect was not observed in SIRT3 knockout mice (81). In addition, the activation of SIRT3 by RSV attenuated collagen deposition and improved cardiac contractile function (81).

Yet another approach to increase SIRT3 activity is by elevating NAD^+^ levels by feeding the NAD^+^ salvage pathway using the NAD^+^ precursor molecule NMN. By administration of NMN, Pillai et al. could attenuate phenylephrine-induced cardiomyocyte hypertrophy in neonatal rat cardiomyocytes and AngII-induced cardiac hypertrophy in mice (29). Importantly, they also demonstrated that NMN treatment attenuated isoproterenol-induced cardiac hypertrophy, a beneficial effect that was blunted in mice with additional SIRT3 deficiency (29). Even more impressive, Lee et al. recently demonstrated that normalization of the NAD^+^ redox balance by NMN treatment or overexpression of the rate-limiting enzyme for NAD^+^ synthesis, nicotinamide phosphoribosyltransferase (Nampt), improved cardiac hypertrophy and dysfunction induced by isoproterenol treatment or TAC, strongly suggesting that NAD^+^ repletion may represent a promising therapeutic approach to treat cardiac hypertrophy and heart failure (82). Similar positive effects of NAD^+^ repletion were observed in the setting of IR, where NAD^+^ is lost during reperfusion (63). Reraising NAD^+^ levels by overexpression of Nampt or by administration of NMN resulted in reduced myocardial infarct size following IR (63, 83). Although none of the above mentioned substances (honokiol, RSV, NMN) is a specific activator of SIRT3, the results are encouraging that specific activation of SIRT3 as a therapeutic strategy may have beneficial effects in heart disease.

## Other Mitochondrial Sirtuins

SIRT4 and SIRT5 are further mitochondrial sirtuins that require NAD^+^ as a cosubstrate and which seem to oppose or complement the function of SIRT3 in the heart. Thus far, only few is known about SIRT4 and SIRT5 in general, compared with SIRT3. While the *in vitro* deacetylation activity of SIRT4 seems to be much lower compared with SIRT3, other posttranslational modifications may be of higher relevance, such as delipoylation and ADP ribosylation, although the latter one may simply represent a side reaction of the main deacylation activity without significant physiological relevance (84–87). SIRT4 has been shown to regulate energy metabolism by inhibiting glutamate dehydrogenase by ADP ribosylation, to inhibit the pyruvate dehydrogenase complex by hydrolyzing the lipoamide cofactors from the E2 component dihydrolipoyllysine acetyltransferase, and to regulate lipid metabolism by deacetylating malonyl-CoA decarboxylase, resulting in malonyl-CoA accumulation and inhibition of FAO (85–87). SIRT4 is highly expressed in the heart, but does not seem to affect the physiologic pump function of the heart when genetically overexpressed or deleted (88). In contrast, increased cardiac hypertrophy and aggravated cardiac dysfunction have been observed in response to AngII treatment in mice with cardiomyocyte-restricted overexpression of SIRT4 (88). Mechanistically, mitochondria-targeted antioxidant treatment prevented the aggravation of cardiac hypertrophy in SIRT4-overexpressing mice, suggesting increased mitochondrial oxidative stress as the cause. The authors proposed that SIRT4 may inhibit binding of SIRT3 to MnSOD, thereby increasing MnSOD acetylation and impairing H_2_O_2_ detoxification (88). Regarding IR injury in the heart, hypoxia downregulated the expression of SIRT4 in H9C2 rat heart myoblasts, and knockdown of SIRT4 decreased cell viability, increased caspase activation, and increased the amount of apoptotic cells, suggesting that downregulation of SIRT4 during hypoxia may contribute to increased apoptosis in response to hypoxia and IR (89). In contrast, SIRT4 siRNA treatment protected against induction of mPTP opening and mPTP-dependent ROS production in another study, leaving the role of SIRT4 in myocardial IR injury incompletely elucidated (90).

SIRT5 also exerts only weak deacetylase activity, but instead catalyzes desuccinylation, demalonylation, and deglutarylation (91, 92). Large-scale proteomic analyses identified many proteins to be putative targets of SIRT5, including enzymes of amino acid degradation, the TCA cycle, FAO, pyruvate decarboxylation, and ATP synthesis (91). SIRT5 is highly expressed in cardiac tissue. Mice with deletion of SIRT5 develop cardiac dysfunction, pathologic hypertrophy, and increased fibrosis (93). Analysis of the cardiac succinylome in SIRT5^−/−^ mice suggested mitochondrial energy metabolic pathways to be regulated by SIRT5, and that myocardial energy depletion may contribute to cardiac dysfunction in SIRT5^−/−^ mice, potentially due in part to hypersuccinylation and inhibition of the FAO enzyme enoyl-CoA hydratase α (93). Lack of SIRT5 also modulates the extent of IR injury. Myocardial infarct size was increased, and recovery of contractile function was impaired in hearts of SIRT5^−/−^ mice following IR (94). IR injury was restored to WT levels by inhibition of succinate dehydrogenase, which was hypersuccinylated and probably more active in SIRT5^−/−^ hearts. Since succinate accumulates during ischemia and drives mitochondrial ROS generation at the onset of reperfusion by increasing reverse electron transport in the respiratory chain, the authors proposed that inhibition of succinate dehydrogenase may restore IR injury to WT levels by reducing succinate-driven ROS production (94, 95).

Taken together, both SIRT4 and SIRT5 may play a role in cardiac pathologies, and more studies are needed to further understand their mechanism of action. Deciphering the role of each mitochondrial sirtuin is important since some of the promising therapeutic approaches, in particular NAD^+^ repletion, should increase the activity of all mitochondrial sirtuins simultaneously.

## Conclusion

Mitochondrial damage and dysfunction contribute to the pathogenesis of different cardiac pathologies, including heart failure and IR injury (30, 31, 62). Impairment in SIRT3 expression and/or activity has been proposed to contribute to these mitochondrial defects by slowing metabolic substrate oxidation, boostering mitochondrial oxidative stress, and increasing the sensitivity for mPTP induction. Based on beneficial effects on cardiac hypertrophy and IR injury by treatment with honokiol, resveratrol, and NMN, which increase the expression and/or activity of SIRT3, the development of specific SIRT3 activators could represent a novel therapeutic strategy by which multiple mitochondrial defects may be treated simultaneously. It needs to be kept in mind though that SIRT3 may interact with other mitochondrial sirtuins (SIRT4, SIRT5) as well; the myocardial functions of which are still poorly understood. Thus, more studies are needed to deepen our understanding of all mitochondrial sirtuins in cardiac physiology and disease. Based on our current knowledge, modulation of mitochondrial sirtuin activity seems to have great therapeutic potential and may advance the field of mitochondrial medicine also for diseases beyond cardiovascular disease.

## Author Contributions

CK wrote the manuscript. CB and HB reviewed and edited the manuscript.

## Conflict of Interest Statement

The authors declare that the research was conducted in the absence of any commercial or financial relationships that could be construed as a potential conflict of interest.
